# A probabilistic approach to tomography and adjoint state methods, with an application to full waveform inversion in medical ultrasound

**DOI:** 10.1088/1361-6420/ac55ee

**Published:** 2022-03-14

**Authors:** Oscar Bates, Lluis Guasch, George Strong, Thomas Caradoc Robins, Oscar Calderon-Agudo, Carlos Cueto, Javier Cudeiro, Mengxing Tang

**Affiliations:** 1 Department of Bioengineering, Imperial College London, SW7 2AZ, United Kingdom; 2 Earth Science and Engineering Department, Imperial College London, SW7 2AZ, United Kingdom

**Keywords:** tomography, Bayesian methods, stochastic variational inference, adjoint method, full-waveform inversion

## Abstract

Bayesian methods are a popular research direction for inverse problems. There are a variety of techniques available to solve Bayes’ equation, each with their own strengths and limitations. Here, we discuss stochastic variational inference (SVI), which solves Bayes’ equation using gradient-based methods. This is important for applications which are time-limited (e.g. medical tomography) or where solving the forward problem is expensive (e.g. adjoint methods). To evaluate the use of SVI in both these contexts, we apply it to ultrasound tomography of the brain using full-waveform inversion (FWI). FWI is a computationally expensive adjoint method for solving the ultrasound tomography inverse problem, and we demonstrate that SVI can be used to find a no-cost estimate of the pixel-wise variance of the sound-speed distribution using a mean-field Gaussian approximation. In other words, we show experimentally that it is possible to estimate the pixel-wise uncertainty of the sound-speed reconstruction using SVI and a common approximation which is already implicit in other types of iterative reconstruction. Uncertainty estimates have a variety of uses in adjoint methods and tomography. As an illustrative example, we focus on the use of uncertainty for image quality assessment. This application is not limiting; our variance estimator has effectively no computational cost and we expect that it will have applications in fields such as non-destructive testing or aircraft component design where uncertainties may not be routinely estimated.

## 1 Introduction: probabilistic tomography and adjoint-methods

### 1.1 The review

Tomographic imaging seeks to render internal anatomical structures visible, a task which is complicated because the quality of a tomographic image is imperfect. Examples of imperfections include blur, artefacts, or a failure to reconstruct altogether [1]. Across the scientific disciplines, uncertainty is the method of choice for quantifying the degree of reliability of a chosen model. We show that, in the case of tomographic imaging, a pixel-wise estimate of uncertainty is capable of measuring image quality objectively and without relying on reference images. Our approach is to apply stochastic variational inference (SVI), a gradient-based approach for solving Bayes’ equation, to full-waveform inversion (FWI), a reconstruction algorithm designed for wavefield data such as ultrasound. FWI uses a full numerical simulation of the propagation of a wavefield through the sample, and solves the inverse problem using an adjoint-state method. Therefore, our method is readily applicable to tomography and adjoint-state methods in other fields.

Image quality can be improved by carefully adjusting the settings of a reconstruction. Settings, such as inversion frequency in FWI, can cause the reconstruction algorithm to fail when they are not tailored correctly. Calibrating an imaging system currently requires extensive clinical guidance and expert judgement [1]. In addition, line-pair phantoms (e.g. R3L3S1N Negative 1951 USAF Target, ThorLabs, NJ, USA) are often used to measure resolution, but this is misleading because the sharpness, and therefore resolution, of the reconstruction changes according to the subject in the image. Uncertainty estimation could mitigate these problems [2]; the uncertainty estimator described herein is sensitive to resolution or blur, reconstruction failure, and artefacts. It is also computationally inexpensive, meaning it can be calculated for every reconstruction and that image quality can be continually monitored. In addition, the uncertainty estimates could be used to automatically re-calibrate imaging apparatus by seeking to minimise the reconstruction variance.

Pixel-wise uncertainty estimates can help to inform clinicians during image analysis, for example knowing the effective resolution of the reconstruction can inform a clinician of the minimum feature size which is relevant. In addition, the advent of machine-led image analysis increases the need for uncertainty estimates because artificial intelligence algorithms lack human-equivalent perception; they can be easily misled by changes in image quality. Uncertainty estimation might be an effective mitigation strategy because acceptable image quality bounds can be stated in a clear and machine-comprehensible format.

Medical image quality has advanced dramatically in recent decades and high-resolution, high-contrast 3D imaging is now available. However clinical decision making based on imagery is imperfect, for example Myers *et al* [3] report that mammographic screening programmes have a cumulative false positive rate of between 4% and 10% and that there is significant variation between clinicians. In part, this is because imperfect image analysis can lead to false results. An alternative approach is quantitative imaging, which provides numerical measurements of specific properties of the tissues under observation. Quantitative imaging simplifies image analysis by providing a directly comparative measurement between patients. Unfortunately, modelling physics approximations made during the design of the reconstruction algorithm can lead to drift between patients. For example, dual energy x-ray absorptiometry, which measures the response of tissues to x-ray beams with different energy profiles, can drift due to changes in the thickness of the sample [4].

Ultrasound computed tomography (USCT) is an emergent 3D tomographic imaging system which has been studied for imaging the breast [5] and brain [6]. Hopp *et al* [7] explore breast tissue classification according to quantitative sound-speed and absorption measurements. Unfortunately the distributions of fatty, glandular and malignant breast tissues overlap, leading to misclassification if a measurement falls on the wrong side of the decision boundary. This is a common problem with datasets that are not linearly separable, and uncertainty helps by providing a range of values that a pixel can reasonably be expected to take. This means that sound speeds close to the decision boundary can be assigned a probability of belonging in either class, and prioritised for more careful assessment.

Tomographic reconstruction algorithms rarely provide uncertainty estimates because pixel-wise uncertainty estimation is computationally expensive. Iterative reconstruction is a commonly used tomographic reconstruction algorithm and has its statistical basis in *maximum likelihood estimation* (MLE), section 2.1 demonstrates MLE in the context of FWI. Fessler and Hero [8] and Fessler [9] use the MLE framework to show that covariance matrix of the reconstruction can be calculated by taking the inverse of the Hessian of the likelihood distribution. Unfortunately, the Hessian is computationally intractable for realistic problems and particularly for 3D tomography [2].

MLE is a frequentist statistical technique. Bayesian methods offer numerous practical advantages and are a popular alternative [10]. Bayesian methods which neglect uncertainty estimation are called *maximum a posteriori* (MAP) estimation. The main difference between MLE and MAP estimation is the use of prior information in the form of a regulariser. The typical regulariser is an additional term in the objective function which penalises reconstructions that do not have favourable properties, this is also called a weak constraint. The simplest example is Tikhonov regularisation, which uses the L2-norm of the parameter vector to penalise the objective. Further details about regularisation can be found in [10].

This paper favours Bayesian inference, which combines regularisation with uncertainty estimation. Bayesian inference is less common than MAP in tomographic reconstruction. One approach to Bayesian inference is to replace the iterative reconstruction with a neural network: the data-to-model approach. Barbano *et al* [11] use deep gradient unrolling, a method which replaces the gradient descent process with a neural network. The network is broken into blocks, where each block corresponds to a single iteration of gradient descent. A block takes a predicted reconstruction and the gradient of the likelihood function as inputs, and uses the neural network to produce the predicted reconstruction for the next block. The whole neural network is trained using a likelihood function, which is the L2-norm of the difference between the predicted reconstruction (the output of the final block) and an iterative reconstruction. Uncertainty estimates are obtained by including a ‘Bayesian convolutional layer’ at the end of each block. Because these layers are stochastic, they will produce a range of possible reconstructions from the deterministic inputs. An alternative approach by Tonolini *et al* [12] could be applicable to medical tomography. A conditional variational auto-encoder is trained to perform reconstructions from corrupted image data. The network is conditioned on the imaging device so that the reconstruction can generalise across different acquisition devices. Variational auto-encoders have a stochastic latent space, so sampling the latent space produces a range of possible reconstructions.

Alternatively, deep-image-prior (DIP) methods use an untrained convolutional network to regularise an iterative reconstruction, thereby using the structure of the convolutional network to favour reconstructions which are visually plausible. Most DIP approaches use MAP estimation. However, Narnhofer *et al* [13] use a Bayesian neural network (BNN) as a DIP. They ensemble several iterative reconstruction processes, each with a different starting point. The starting points are generated by taking independent samples from the DIP generator, meaning that pixel-wise mean and uncertainty estimates can be determined from the mean and variance of the numerous final reconstructions. There is a compromise inherent in this proposal; to determine the mean and uncertainty accurately a large number of reconstructions are required, but a large number of reconstructions mean that computation time will increase linearly because each reconstruction process is independent. Thus the ensembling approach becomes computationally expensive when a physically expressive algorithm such as FWI is used.

Neural-network-based methods can produce excellent reconstructions and they have fast uncertainty estimation, but such data-to-model approaches are sensitive to small changes in the data [14]. Further issues with the data-to-model approach include the large memory footprint of neural networks (which could exclude 3D tomographic reconstruction), a need for large paired training datasets, and poor generalisation across imaging devices. Most concerning is that models designed around neural networks can hallucinate features into the reconstruction [15]. Hallucination seems to be most significant for the DIP approach, suggesting that training can mitigate this problem.

Finally, Markov chain Monte-Carlo (MCMC) algorithms are a popular class of methods to solve Bayesian inference. Unfortunately, MCMC has a comparable computational cost to Hessian-based approaches and their use is restricted to geophysical tomography [16, 17], which has fewer time constraints. A potentially cheaper technique is the stochastic gradient Langevin dynamics algorithm, which has been applied to positron emission tomography reconstruction [18]. Stochastic gradient Langevin dynamics couples stochastic gradient descent to a normally distributed Monte-Carlo sampler. The step-size of the stochastic gradient is reduced as the iterations progress so that the Monte-Carlo sampling term dominates the final set of reconstructions. The mean and uncertainty are calculated from this final set of reconstructions. Unfortunately this algorithm is challenging to implement because it is difficult to specify an appropriate step-size schedule. Thus, it is practically difficult to tune the step-size schedule and the optimal schedule is believed to have slower asymptotic convergence than some MCMC solvers [19].

### 1.2 The claim

This paper contains two distinct parts. The first part (sections 2 and 3) is a theoretical introduction to SVI and its application to FWI. SVI–FWI consists of Bayesian model which produces a MAP estimator, that is equivalent to the standard FWI estimator, and an additional uncertainty estimate, that can be calculated with little additional computational cost. More sophisticated approximations of the posterior are possible, albeit with greater computational cost. Thus, SVI–FWI approach produces two images, an image reconstruction that is comparable to the standard FWI approach and a variance reconstruction which provides a pixel-wise estimate of the variance. The SVI approach that we describe is not limited to FWI and can be applied to other tomographic reconstruction algorithms or adjoint methods.

The second part (section 4) is an illustrative example of the qualitative use of the mean-field Gaussian estimator for image quality assessment. An *in vitro* study shows that the reconstruction variance reduces as the sharpness or resolution of the reconstructed image improves, we call this effect progressive decoding. This study also finds the reconstruction variance is sensitive to reconstruction failure caused by cycle-skipping [20], and an artefact caused by source-position error. In addition, an *in silico* study shows the sensitivity of the reconstruction variance to discrepancies between the physics of some simulated wavefield data and the reduced physics of the inversion algorithm.

### 1.3 The agenda

In the theory (section 2), an introduction to frequentist and Bayesian methods is presented in the context of FWI. Initially, this focuses on the theory of the frequentist *MLE* approach. This is the traditional, statistical basis for iterative tomographic reconstruction. We emphasise that minimising a data-fit function with the form of an L2-norm is statistically equivalent to maximising a likelihood function with a Gaussian form. Then, the Bayesian SVI method is described in detail, with emphasis on the choice between the score-function or the reparameterisation approach. Finally, in the method (section 3), the reparameterisation approach is applied in FWI using a mean-field Gaussian approximation to describe the pixel-wise distribution of the reconstructed image. The application of mean-field SVI to FWI produces an update for the mean which is equivalent to the standard FWI estimator, and an update for the variance which provides uncertainty with no additional computational cost. In section 4, variance reconstructions are produced using experimental and numerical examples, and their application to image quality assessment is demonstrated. This is followed, in section 5, by a discussion about the results, the utility of the estimator, and some directions for further research.

## 2 Theory: statistical interpretations of tomography

This section presents a technical introduction to two statistical methods; *MLE*, which is the basis of traditional reconstruction, and SVI, which is relevant to the development of our method in section 3. In addition, our discussion of MLE and SVI will contextualise their use in tomography and adjoint methods. The link between iterative tomographic reconstruction and MLE has been explored by others [8, 9], but this interpretation is not explicitly discussed in the FWI literature. Furthermore, although iterative reconstruction has become popular across tomography, uncertainty estimation has not achieved widespread use due to the computational cost involved in estimating the Hessian of the likelihood function.

Our specific interest is FWI for 2D and 3D ultrasound tomography. Ultrasound tomography seeks to invert observed wavefield data to obtain a pixel-wise estimate of the sound-speed of an object. Observed wavefield data is produced by measuring pressure changes on the surface of a piezo-electric transducer, herein referred to as a receiver. The pressure wavefield originates at a source (also a piezo-electric transducer), which produces a pressure disturbance in its surroundings. The disturbance propagates away from the source and is modulated by a sample, or patient, before it arrives at the receiver. In other applications the sources and receivers can use different methods to produce and record wavefield disturbances, this can include recording displacement rather than pressure.

### 2.1 The maximum likelihood interpretation of FWI

FWI is a reconstruction algorithm that uses a model of the physics to simulate a wavefield. A popular choice of physics model is the acoustic wave-equation, whose operator □(*c*) is defined,

(1) $$□(c)=\left[c^{-2}\frac{\partial^{2}}{\partial t^{2}}-\nabla^{2}\right].$$

This is also known as the d’Alembert operator. The acoustic wave-equation operator is dependent on a set of acoustic sound speeds *c*, which are usually time-constant but spatially inhomogeneous.

In general, the ground-truth physics is modelled using a partial differential equation operator *H*(·) with model parameters *m*. Together, the operator, the model parameters, and a source disturbance *ς*(*x*_s_, *t*), can be solved to simulate the propagation of a wavefield,

(2) $$H(m)u_{\text{p}}\left(x,t;m,x_{\text{s}},\varsigma \right)=\varsigma \left(x_{\text{s}},t\right),\;$$

where the simulated wavefield *u*_p_(*x, t*) is commonly referred to as the predicted wavefield.

In the case of ultrasound tomography, the source disturbance is the time-varying pressure on the surface of the piezo-electric transducer. Clearly, the source disturbance is non-zero only at the source positions *x*_s_. Similarly, the wavefield cannot be measured throughout the imaging region. Instead, predicted wavefield data *d*_p_(*x*_r_, *t*) are extracted from the wavefield *u*_p_(*x, t*) at the receiver positions *x*_r_ such that,

(3) $$d_{\text{p}}\left(x_{\text{r}},t;m,x_{\text{s}},\varsigma \right)=u_{\text{p}}\left(x_{\text{r}},t;m,x_{\text{s}},\varsigma \right).$$

Thus equation (2) is a time dependent map from the model parameters to the wavefield data. This means a further model can be defined,

(4) $$L\left(m;t,x_{\text{r}},x_{\text{s}},\varsigma \right)=d_{\text{p}}\left(x_{\text{r}},t;m,x_{\text{s}},\varsigma \right)$$

where *L*(*m*) is a non-linear predictor, which maps the model parameters to the predicted wavefield data for a particular time position, receiver position, source position, and source disturbance. In practice, a numerical simulator (for example, finite-difference, finite-element, etc [21]) can be used to solve equations (2) and (3) so that predicted wavefield data are simulated from a set of model parameters.

The predictor can estimate the observed wavefield data *d*(*x*_r_, *t, x*_s_, *ς*) such that,

(5) d=L(m)+γ

which differs from the output of the predictor by an error *γ*. Note that the fixed parameters have been dropped for compactness. By a simple rearrangement we obtain,

(6) γ=d−L(m).

Now, consider an experiment where a pressure disturbance is produced at a source. The disturbance propagates from the source to the receivers, and is modulated by the parameters of the materials between them. The observed wavefield data *d*(*x*_r_, *t*) is the measurement of the pressure at the receivers. In this example, the model parameters are fixed, but other uncontrolled variables might alter the measurement in a random manner. Thus, the observed wavefield data *d* is a random function of the fixed model parameters *m* of the sample. Assuming the model parameters *m* are known and that the error follows a Gaussian distribution, then equation (6) can be used to define a probability distribution *p*(𝒟|*m*) with mean 0 and variance Σ^2^, where 𝒟is the set of all the measurements of the observed wavefield data 𝒟 = {*d*}.

A different situation arises when solving the inverse problem. In this case, the model parameters are varied in order to maximise the estimated probability of some fixed observed wavefield data. Thus, the output is no longer a true estimate of the probability of the data because the true model parameters are unknown. However, the probability distribution of the error provides the structure for the relationship between the observed wavefield data and the model parameters. Hence, equation (6) is used to define a deterministic function ℒ(*m*|𝒟) which describes the likelihood of the model parameters given the data. Assuming the true error is Gaussian distributed, then the likelihood function for a particular model parameter estimate is,

(7) $$ℒ(m|D)=\text{det}{\left(2\pi \sum^{2}\right)}^{-\frac{1}{2}}\text{exp}\left\{-\frac{1}{2}\left[\gamma^{\text{T}}\sum^{-2}\gamma \right]\right\}$$

and, by inserting the definition of the error from equation (6),

(8) $$ℒ(m|D)=\text{det}{\left(2\pi \sum^{2}\right)}^{-\frac{1}{2}}\text{exp}\left\{-\frac{1}{2}{\left[d-L(m)\right]}^{\text{T}}\sum^{-2}[d-L(m)]\right\}.$$

The maximum of the likelihood function is found by optimisation with respect to the model parameters such that,

(9) $$\hat{m}=\text{arg}\;\underset{m}{\text{max}}\;\text{det}{\left(2\pi \sum^{2}\right)}^{-\frac{1}{2}}\text{exp}\left\{-\frac{1}{2}{\left[d-L(m)\right]}^{\text{T}}\sum^{-2}[d-L(m)]\right\},$$

where $\hat{m}$ is optimum set of model parameters, also known as the maximum likelihood estimator.

As the equation (7) has the form *f* (*γ*) = exp(−*γ*^T^*γ*), the magnitude of the function decreases as the magnitude error increases, meaning that an equivalent but numerically simpler process is to maximise the data-fit directly,

(10) $$\hat{m}=\text{arg}\;\underset{m}{\text{max}}-\frac{1}{2}{\left[d-L(m)\right]}^{\text{T}}[d-L(m)],$$

where it is also assumed that Σ^2^ = *I* and *I* is the identity matrix.

This data-fit function is a popular choice for FWI [20, 22, 23], and the same process can be followed for any system described by a physical model *L*(*m*). Thus, we have demonstrated that many FWI approaches assume the observed wavefield data are Gaussian distributed.

The most important implication of this section is to justify the adoption a Bayesian likelihood with a Gaussian form in section 3.2. We have demonstrated that this choice is consistent with the approximations adopted for traditional iterative reconstruction. Furthermore, a no-cost uncertainty estimator can be found using the Bayesian setting. This is in contrast to previous work, which has used the Hessian of the likelihood function to estimate variances associated with tomography. Obtaining variance via the Hessian is consistent with an MLE interpretation of tomography as these variance estimates are found using the Cramer–Rao lower bound [8, 9]. However, Hessian based approaches are too numerically expensive to achieve widespread use.

### 2.2 The variational Bayesian interpretation of FWI

Variational inference is a method to solve Bayes’ equation by gradient-based optimisation. Because the MLE approach also uses gradient-based optimisation, variational inference is more comparable to the MLE-based approach than alternative Bayesian methods such as Monte-Carlo sampling. To introduce variational inference, we start by deriving the evidence lower bound (ELBO), which underlies all variational inference. Finally, we discuss the two solutions of the ELBO which encompass SVI.

Variational inference is a Bayesian method. In the context of FWI, Bayes’ equation can be written as,

(11) $$p(m|D)=\frac{p(D|m)p(m)}{p(D)},$$

where the four terms present are: the posterior distribution *p*(*m*|𝒟), the prior distribution *p*(*m*), the evidence distribution *p*(𝒟), and a conditional distribution *p*(𝒟|*m*) which can be interpreted as a likelihood function for fixed 𝒟.

Previously, we stated that MLE seeks to obtain a point-estimate $\hat{m}$ at the optimum of the likelihood function. In contrast, Bayesian methods seek to obtain a distribution, called the posterior distribution *p*(*m*|𝒟), which describes the distribution of a set of model parameters *m* based on a set of observed measurement data 𝒟 = {*d*}. Moments (i.e. mean, variance) can be obtained directly from the posterior distribution, which means that, in comparison to MLE, Bayesian methods can obtain additional information from the measurement data without requiring the computationally expensive Hessian. Similar to the previous section, *p*(𝒟|*m*) can be interpreted as a likelihood function for fixed 𝒟. Section 3 demonstrates that this likelihood function can also use a Gaussian approximation. The prior distribution *p*(*m*) describes the expected distribution of the model parameters before any data is observed. When multiplied together using the law of total probability, the prior and likelihood are collectively called the joint distribution *p*(*m*, 𝒟) such that *p*(*m*, 𝒟) = *p*(𝒟|*m*)*p*(*m*). The evidence is the marginal of the joint distribution, which is defined symbolically below.

The aim of Bayesian methods is to obtain an estimate of the posterior distribution using known estimates of the likelihood, prior and evidence. This is difficult to achieve for most inverse problems because the number of model parameters means the evidence is numerically intractable. To illustrate this, consider that the evidence is obtained by marginalising the joint distribution *p*(𝒟, *m*) over the model parameters,

(12) $$p(D)=\int_{m}p(D,m)\text{d}m$$

which can be rearranged using the law of total probability,

(13) $$p(D)=\int_{m}p(D|m)p(m)\text{d}m.$$

The numerical integration, which is required to solve equation (13), is intractable due to the number of combinations of model parameters, which also means the posterior is intractable. Exceptions occur when the prior and likelihood have analytical expressions which are conjugate (i.e. they come from related distributional families, for example, when both distributions are Gaussian). In this case, analytical solutions to Bayes’ equation can be found which are relatively inexpensive to invert, such as by a Gaussian process.

Another class of solvers are Monte-Carlo methods which sample the posterior directly such that,

(14) $$q_{\text{MC}}(m|D)=\frac{1}{C}\sum_{i}^{N}p\left(D|m^{\left(i\right)}\right)p\left(m^{\left(i\right)}\right),$$

where *q*_MC_(*m*|𝒟) is the Monte-Carlo estimate of the posterior, *p*(*m*^(*i*)^) ~ *p*(*m*) is a sample from the prior distribution, and *C* is a normalising constant. Thus, samples from the prior distribution *p*(*m*) are used to calculate the joint estimate *p*(*m*, 𝒟), from which the Monte-Carlo approximation to the posterior can be calculated directly by summing over multiple samples. Note that the evidence distribution has been replaced by the normalising constant *C*, which simply scales the output distribution into the range between 0 and 1. As the number of samples increases, the approximate Monte-Carlo posterior *q*_MC_(*m*|𝒟) will converge to the true posterior *p*(*m*|𝒟). Monte-Carlo methods can be effective, but they are fundamentally limited because they need at least tens of samples per parameter. This makes Monte-Carlo methods computationally expensive, and often intractable, for high dimensional problems such as tomography.

An alternative approach is variational inference, which tries to solve Bayes’ equation using gradient-based optimisation methods. Variational inference seeks to compare an approximating distribution *q*(*m*) to the posterior distribution *p*(*m*|𝒟) by using the Kulback–Leibler (KL) divergence,

(15) $$KL[q(m)∥p(m|D))]=\int_{m}q(m)\ln\left[\frac{q(m)}{p(m|D)}\right]\text{d}m.$$

At this point the approximating distribution has no specific form, for example it could be parametric or non-parametric, mono-modal or multi-modal. Later, we will make some assumptions about the structure of the distribution to make the problem tractable. For now, we note that the optimal approximate distribution q*(*m*) is found at the minimum of the KL divergence,

(16) $$q^{*}(m)=\underset{q(m)}{\text{min}}\;KL[q(m)∥p(m|D))].$$

This formulation leaves two unsolved problems:

1. The posterior *p*(*m*|𝒟), as mentioned above, is intractable.
2. Performing gradient descent with respect to a distribution (i.e. the derivative with respect to the approximating distribution *q*(*m*)) is not well defined.

#### 2.2.1 Problem I

The ELBO, commonly referred to as the ELBO, provides the solution to problem (a). To find the ELBO, start by using Bayes’ equation *p*(*m*|𝒟) = *p*(*m*, 𝒟)/*p*(𝒟) to split the posterior in equation (15) such that,

(17) $$KL[q(m)∥p(m|D))]=\text{ln}\;p(D)+\int_{m}q(m)\text{ln}\left[\frac{q(m)}{p(m,D)}\right]\text{d}m.$$

Note that the logarithm of the evidence distribution is always negative because probabilities are bounded from 0 to 1 and that, by definition, the KL-divergence is positive;

(18) KL[q(m)∥p(m|D))]⩾0.

Therefore, by applying this definition to equation (17), we have,

(19) lnp(D)⩾−KL[q(m)∥p(m,D))]

which defines the ELBO. Finally, observe from equation (17) that ln *p*(𝒟) = −𝕂𝕃[*q*(*m*)∥*p*(*m*, 𝒟)] if, and only if, 𝕂𝕃[*q*(*m*)∥*p*(*m*|𝒟))] = 0. Therefore, the maximum of the ELBO occurs if, and only if, the approximating distribution is equal to the posterior distribution. Thus, an new optimisation problem (which is equivalent to equation (16)) is obtained such that,

(20) $$\begin{array}{l} q^{*}(m)=\underset{q(m)}{\text{max }}KL[q(m)∥p(m,D)]\\ \;=\underset{q(m)}{\text{max}}\int_{m}q(m)\ln\left[\frac{q(m)}{p(m,D)}\right]\text{d}m. \end{array}$$

Because this KL-divergence is taken with respect to the joint distribution *p*(*m*, 𝒟) rather than the posterior *p*(*m*|𝒟), any explicit dependence on the evidence distribution *p*(𝒟) has been removed from the optimisation problem. Therefore, the marginalisation of the joint distribution (from equation (13)) is no longer required and we find that equation (20) can be solved numerically. Section 3 deals with the practical aspects of specifying each distribution such that they are tractable for FWI, meaning that we can conclude the discussion of problem (a).

#### 2.2.2 Problem II

There are several approaches to solve problem (b). Two of these approaches are classed as SVI and seek to replace the approximating distribution *q*(*m*) with a parametric equivalent *q*(*m*; λ), where λ are a set of parameters describing the distribution. This means that the optimisation problem can be posed to optimise the ELBO with respect to the parameters of the approximating distribution rather than the distribution itself. Therefore, consider that we can choose a parametric approximating distribution *q*(*m*; λ) (e.g. the binomial distribution, the Gaussian distribution, etc), and adjust equation (20) such that,

(21) $$\begin{array}{l} q^{*}(m|\lambda )=\text{arg}\;\underset{\lambda }{\text{max}}\;KL[q(m;\lambda )∥p(m,D)]\\ \;=\text{arg}\;\underset{\lambda }{\text{max}}\int_{m}q(m;\lambda )\ln\left[\frac{q(m;\lambda )}{p(m,D)}\right]\text{d}m. \end{array}$$

The aim is to update the approximating distribution using the gradient of the KL-divergence in equation (21). Both of the SVI methods discussed below will obtain the gradient by Monte-Carlo estimation but, in contrast to direct Monte-Carlo estimation of the posterior (i.e. equation (14)), we will use a Monte-Carlo method to estimate the gradient of the ELBO (as defined in equation (21)). Monte-Carlo methods sample from a distribution so that the approximate moments of the distribution can be estimated. In this case, the Monte-Carlo method allows the first moment (i.e. the expectation or mean) of the ELBO to be estimated.

To obtain the Monte-Carlo estimate of the gradient, first it is necessary to reinterpret equation (21) as an expectation such that,

(22) $$KL[q(m;\lambda )∥p(m,D)]=E_{q(m;\lambda )}\left[\text{ln}\left\{\frac{q(m;\lambda )}{p(m,D)}\right\}\right].$$

Thus, a gradient of the ELBO can be taken with respect to the parameters λ of the optimisation,

(23) $$\nabla_{\lambda }E_{q(m;\lambda )}\left[\ln\left\{\frac{q(m;\lambda )}{p(m,D)}\right\}\right]=\nabla_{\lambda }\int_{m}q(m;\lambda )\text{ln}\left\{\frac{q(m;\lambda )}{p(m,D)}\right\}\text{d}m.$$

Superficially, it appears that we can calculate the gradient of the ELBO by taking the expectation of the gradient of the term in the logarithm such that,

(24) $$\begin{array}{l} E_{q(m;\lambda )}[\nabla_{\lambda }\text{ln}\{\frac{q(m;\lambda )}{p(m,D)}\}]=\int_{m}q(m;\lambda )\nabla_{\lambda }\text{ln}\{\frac{q(m;\lambda )}{p(m,D)}\}\text{d}m\\ \;\neq \nabla_{\lambda }E_{q(m;\lambda )}[\ln\{\frac{q(m;\lambda )}{p(m,D)}\}]. \end{array}$$

Unfortunately, it is apparent that this operation is not valid because of the dependence of the expectation on the approximating distribution itself. This means that the estimator on the right-hand side of equation (24) is biased relative to the estimator defined in equation (23). In other words, a gradient of the ELBO with respect to the parameters of the approximating distribution ∇_λ_ cannot be exchanged with the expectation term, because the expectation is implicitly dependent on an integrand which contains the approximating distribution such that 𝔼_*q*(*m*;λ)_ [*f*(*m*)] = ⎰ *q*(*m*; λ) *f*(*m*)d*m*.

Alternatively, the score function approach [24] rearranges equation (22) to find an unbiased estimator of the ELBO. Using the chain rule and a method called the log-derivative trick, in which the relationship ∇_*x*_ *f* (*x*) = *f* (*x*) ∇_*x*_ ln[*f* (*x*)] is used, we obtain an unbiased estimate of the gradient of the ELBO,

(25) $$\begin{array}{l} \nabla_{\lambda }E_{q(m;\lambda )}\left[\ln\left\{\frac{q(m;\lambda )}{p(m,D)}\right\}\right]=\int_{m}q(m;\lambda )\nabla_{\lambda }\text{ln}\left\{\frac{q(m;\lambda )}{p(m,D)}\right\}\text{d}m\\ \;+\int_{m}q(m;\lambda )\ln\left\{\frac{q(m;\lambda )}{p(m,D)}\right\}\nabla_{\lambda }\ln\{q(m;\lambda )\}\text{d}m\\ \;=E_{q(m;\lambda )}\left[\nabla_{\lambda }\text{ln}\left\{\frac{q(m;\lambda )}{p(m,D)}\right\}\right]\\ \;+E_{q(m;\lambda )}\left[\text{ln}\left\{\frac{q(m;\lambda )}{p(m,D)}\right\}\nabla_{\lambda }\text{ln}\{q(m;\lambda )\}\right]. \end{array}$$

Because the estimator in equation (25) is unbiased, we can take a Monte-Carlo estimate of the gradient. Thus, the approximating distribution is sampled so *q*(*m*^(*i*)^; λ) ~ *q*(*m*; λ), and the integral in equation (25) can be replaced with an asymptotically equivalent operation, namely the discrete sum over the samples, such that,

(26) $$\begin{array}{l} \nabla_{\lambda }E_{q(m;\lambda )}\left[\text{ln}\left\{\frac{q(m;\lambda )}{p(m,D)}\right\}\right]\approx \frac{1}{N}\sum_{i}^{N}\nabla_{\lambda }\text{ln}\left\{\frac{q\left(m^{\left(i\right)};\lambda \right)}{p\left(m^{\left(i\right)},D\right)}\right\}\\ \;+\frac{1}{N}\sum_{i}^{N}\text{ln}\left\{\frac{q\left(m^{\left(i\right)};\lambda \right)}{p\left(m^{\left(i\right)},D\right)}\right\}\nabla_{\lambda }\text{ln}\left\{q\left(m^{\left(i\right)};\lambda \right)\right\}. \end{array}$$

Unfortunately, the score function estimator has high variance because the probabilities are bounded from 0 to 1 and the logarithm tends to infinity when its argument tends to zero, a situation which occurs in the first part of the second expectation in equation (26). This can slow the convergence of the algorithm because the direction of successive gradient estimates becomes unstable.

An alternative approach is to use the reparameterisation trick [25–28]. During reparameterisation, the parametric approximating distribution *q*(*m*; λ) is transformed into a deterministic function *g*(·) of another distribution *q*(*ϵ*) with parameters {λ} such that,

(27) q(m;λ)=g[{λ},q(ϵ)].

As an example, consider a Gaussian distribution where the approximating distribution 𝒩 (*µ*, Σ) can be transformed into the standard normal distribution 𝒩(0, 1) using,

(28) $$\begin{array}{l} N(\mu ,\sum )=g[\{\mu ,\sum \},N(0,1)]\\ \;=\left[\sum^{\text{T}}N(0,1)\right]+\mu , \end{array}$$

where the parameters of the transform λ are the mean vector *µ* and standard deviation matrix Σ. The reparameterisation trick means that the gradient of the ELBO can be taken with respect to the parameters of the transform,

(29) $$\nabla_{\lambda }E_{q(m;\lambda )}\left[\ln\left\{\frac{q(m;\lambda )}{p(m,D)}\right\}\right]=\nabla_{\lambda }\int_{\epsilon }q(\epsilon )\text{ln}\left\{\frac{g[\lambda ,\epsilon ]}{p(g[\lambda ,\epsilon ],D)}\right\}\text{d}\epsilon ,$$

where the shorthand *g*[{λ}, *q*(*ϵ*)] ≡ *g*[λ, *ϵ*] is used. Splitting the expression into a regularisation term and a likelihood term gives,

(30) $$\begin{array}{l} \nabla_{\lambda }E_{q(m;\lambda )}\left[\ln\left\{\frac{q(m;\lambda )}{p(m,D)}\right\}\right]=\nabla_{\lambda }\int_{\epsilon }q(\epsilon )\text{ln}\left\{\frac{g[\lambda ,\epsilon ]}{p(g[\lambda ,\epsilon ])}\right\}\text{d}\epsilon \\ \;-\nabla_{\lambda }\int_{\epsilon }\text{ln}\{p(D|g[\lambda ,\epsilon ])\}\text{d}\epsilon \end{array}.$$

By rearranging the expression, taking the derivative through the integrand, and using the chain rule, we find,

(31) $$\begin{array}{l} \nabla_{\lambda }E_{q(m;\lambda )}\left[\text{ln}\left\{\frac{q(m;\lambda )}{p(m,D)}\right\}\right]=\int_{\epsilon }q(\epsilon )\nabla_{g}\ln\{g[\lambda ,\epsilon ]\}\nabla_{\lambda }g[\lambda ,\epsilon ]\text{d}\epsilon \\ \;-\int_{\epsilon }q(\epsilon )\nabla_{g}\ln\{p(g[\lambda ,\epsilon ])\}\nabla_{\lambda }g[\lambda ,\epsilon ]\text{d}\epsilon \\ \;-\int_{\epsilon }q(\epsilon )\nabla_{g}\ln\{p(D|g[\lambda ,\epsilon ])\}\nabla_{\lambda }g[\lambda ,\epsilon ]\text{d}\epsilon \end{array}$$

and, finally, taking a Monte-Carlo estimate with respect to the new distribution,

(32) $$\begin{array}{l} \nabla_{\lambda }E_{q(m;\lambda )}[\text{ln}\{\frac{q(m;\lambda )}{p(m,D)}\}]=\frac{1}{N}\overset{N}{\underset{i}{\sum }}\nabla_{g}\;\text{ln}\{g[\lambda ,\epsilon_{i}]\}\nabla_{\lambda }g[\lambda ,\epsilon_{i}]\text{d}\epsilon \\ \;-\frac{1}{N}\overset{N}{\underset{i}{\sum }}\nabla_{g}\text{ln}\{p(g[\lambda ,\epsilon_{i}])\}\nabla_{\lambda }g[\lambda ,\epsilon_{i}]\text{d}\epsilon \\ \;-\frac{1}{N}\overset{N}{\underset{i}{\sum }}\nabla_{g}\text{ln}\{p(D|g[\lambda ,\epsilon_{i}])\}\nabla_{\lambda },g[\lambda ,\epsilon_{i}]. \end{array}$$

This is called a pathwise gradient estimator. It is less flexible than the score function estimator, because it requires a parametric transform from a standard distribution, but in section 3 the pathwise estimator is used because it is faster to converge and has lower variance.

## 3 Method: low-cost uncertainty estimation for inverse problems

Variational inference allows us to avoid the computationally intractability of the evidence by arranging Bayes’ equation (equation (11)) such that it can be solved by specifying only the prior distribution, approximating distribution, and likelihood function. It is practical to parameterise these distributions in a number of ways, but the simplest statistical model is to assume that all of the distributions have mean-field Gaussian form. This means that the distributions are easy to specify and solve, but also that they won’t provide a true estimate of the variance because the mean-field Gaussian ignores correlation between the pixels. The subtleties of this approximation are discussed in section 5.

Below, we present the mean-field Gaussian solution for FWI which can be re-purposed to solve other forms of tomography and adjoint methods with limited effort. The mean-field Gaussian produces a mean update, which is similar to standard FWI (for a summary of standard FWI see section 2.1), and a variance update, which has no additional computational cost compared to standard FWI. This proof is the main contribution of this paper. Due to the similarity between standard (MLE) FWI and SVI–FWI, we expect this finding will also generalise to other methods.

### 3.1 An optimisation perspective

It is common to interpret FWI as a local optimisation process with respect to an objective function, where the objective function includes data-fit terms and excludes regularisation terms [20]. Any correct Bayesian interpretation of FWI must include a consideration of prior information and would use regularisation. However, correctly incorporating prior information is complex and problem specific [10], which would add additional complexity to this work. Instead we will consider the correct incorporation of prior information in future work. Therefore, it is expedient to consider the estimation of the parameters of the approximating distribution purely as an optimisation process. Rewriting equation (22) such that,

(33) $$E_{q(m;\lambda )}\left[\text{ln}\left\{\frac{q(m;\lambda )}{p(m,D)}\right\}\right]=-\int_{\epsilon }q(\epsilon )\text{ln}\{p(D|m)\}\text{d}\epsilon +\int_{\epsilon }q(\epsilon )\text{ln}\left\{\frac{q(m;\lambda )}{p(m)}\right\}\text{d}\epsilon$$

it is clear [10] that this expression is equivalent to an optimisation with an objective function of the form,

(34) $$E_{q(m;\lambda )}\left[\text{ln}\left\{\frac{q(m;\lambda )}{p(m,D)}\right\}\right]=-ℒ(m)+a𝒮(m),$$

where the constant *a* has been introduced. This constant modulates the importance of the data-fit term ℒ relative to the regularisation term 𝒮. In practice, the constant is adjusted as a hyper-parameter of the optimisation process. In our analysis we choose *a* = 0, which is a common choice for FWI [20]. Therefore, the full gradient expression, shown in equation (32), can be simplified to,

(35) $$\nabla_{\lambda }E_{q(m;\lambda )}\left[\ln\left\{\frac{q(m;\lambda )}{p(m,D)}\right\}\right]=-\int_{\epsilon }q(\epsilon )\nabla_{g}\text{ln}\{p(D|g[\lambda ,\epsilon ])\}\nabla_{\lambda }g[\lambda ,\epsilon ]\text{d}\epsilon \cdot$$

In the following, equation (35) is used as the starting point for the calculation of the gradients with respect to the mean and standard deviation. This will produce solutions for the approximate posterior which are strictly incorrect, but these solutions are illustrative of the broader technique and have some useful properties.

### 3.2 The mean-field Gaussian approximation

Starting with the prior distribution *p*(*m*), assuming it has a mean-field Gaussian parameterisation, and using equation (28) we find,

(36) $$p(m)=\left[\sum_{\text{prior }}^{\text{T}}q(\epsilon )\right]+\mu_{\text{prior}}.$$

The prior information is not used for regularisation and only to start the reconstruction process. In our case, the inversions start from a mean-field Gaussian prior with mean 1480(m s^−1^) and variance 4 (m s^−1^)^2^.

The prior distribution is a special case of the approximating distribution. In general the approximating distribution is,

(37) $$q(m;\lambda )=\left[\sum^{\text{T}}q(\epsilon )\right]+\mu ,$$

where λ = {*µ*, Σ} are the variational parameters and *q*(*ϵ*) = 𝒩 (0, *I*) is a vector whose elements are samples from the standard normal distribution.

Now, only the likelihood distribution remains to be specified. When describing standard (MLE) FWI, we demonstrated that a data-fit function using the L2-norm is equivalent to a Gaussian likelihood function. Therefore, we will make a comparable choice and use a standard normal Gaussian likelihood function, which leads to the expression,

(38) $$\text{ln}\{p(D|g[\lambda ,\epsilon ])\}=\text{ln}\left(\text{trace}{\left(2\pi I\right)}^{-\frac{1}{2}}\text{exp}\left\{-\frac{1}{2}{\left[d-L(m)\right]}^{\text{T}}[d-L(m)]\right\}\right).$$

The exponential term in the definition of the Gaussian cancels with the logarithm and we obtain the data-fit,

(39) $$\text{ln}\{p(D|g[\lambda ,\epsilon ])\}=\frac{1}{2}{\left[d-L(g[\lambda ,\epsilon ])\right]}^{\text{T}}[d-L(g[\lambda ,\epsilon ])],$$

where *q*(*m*; λ) = *g*[λ, *ϵ*] using the reparameterisation trick. The variational inference likelihood is still a function of the model parameters, but the model parameters are specified by the approximating distribution and, in turn, the approximating distribution is a deterministic function of the two variational parameters. For acoustic FWI, the model parameters are the acoustic sound speed *m* = *c* discretised into pixels and, therefore, the approximating distribution is over the acoustic sound-speed of each pixel.

Together, these functions describe a statistical model which can be solved by taking pathwise derivatives of the likelihood function with respect to the parameters of the mean-field Gaussian approximating distribution.

#### 3.2.1 Gradient with respect to the mean

Taking equation (35) the update for the mean parameter is,

(40) $$\begin{array}{c} \frac{\partial }{\partial \mu }\ln p(D|g[\lambda ,\epsilon ])=\frac{\partial }{\partial g}\ln p(D|g[\lambda ,\epsilon ])\frac{\partial }{\partial \mu }g[\lambda ,\epsilon ]\\ \;=\frac{1}{2}\frac{\partial }{\partial g}{\left[d-L(g)\right]}^{\text{T}}[d-L(g)]\frac{\partial }{\partial \mu }\left(\mu +\sum^{\text{T}}q(\epsilon )\right) \end{array}.$$

Then, using the numerator form of the multi-variable chain $\frac{\text{d}u^{\text{T}}v}{\text{d}m}=u^{\text{T}}\frac{\text{d}v}{\text{d}m}+v^{\text{T}}\frac{\text{d}u}{\text{d}m}$ , and the relationship *A*^T^*B* = *B*^T^*A*, we obtain,

(41) $$\frac{\partial }{\partial \mu }p(D|g[\lambda ,\epsilon ])={\left(\frac{\partial L}{\partial g}\right)}^{\text{T}}[d-L(g)].$$

Finally, taking a sample from the approximating distribution *m*^*i*^ ~ *g*[λ, *ϵ*] means,

(42) $$\frac{\partial }{\partial \mu }p\left(D|m^{i}\right)={\left(\frac{\partial L}{\partial m^{i}}\right)}^{\text{T}}\left[d-L\left(m^{i}\right)\right].$$

Therefore, the iterative process to obtain the mean is,

(43) $$\mu =\mu -{\text{Δ}}_{m}.$$

where ${\text{Δ}}_{m}=\frac{\partial }{\partial \mu }p\left(D|m^{i}\right).$

Note that by assuming *q*(*ϵ*) = 0, then *g*(λ, *ϵ*) = *µ*. Thus, the standard, deterministic, FWI algorithm is returned from the mean update when *q*(*ϵ*) = 0. In addition, the Monte-Carlo sample *m*^*i*^ is not a probability (i.e. bounded from 0 to 1) because we are not dealing directly with the probability distribution *q*(*m*; λ) = *g*[λ, *ϵ*]. Instead, the function *g*[λ, *ϵ*] is used to obtain the sound-speed estimate, which corresponds to a probability *q*(*ϵ*^*i*^) ~ *q*(*ϵ*).

#### 3.2.2 Gradient with respect to the standard deviation

Similarly, we find the standard deviation by taking the derivative with respect to the standard deviation parameter Σ,

(44) $$\begin{array}{c} \frac{\partial }{\partial \sum }\ln p(D|g[\lambda ,\epsilon ])=\frac{\partial }{\partial g}\ln p(D|g[\lambda ,\epsilon ])\frac{\partial }{\partial \sum }g[\lambda ,\epsilon ]\\ \;=\frac{1}{2}\frac{\partial }{\partial g}{\left[d-L(g)\right]}^{\text{T}}[d-L(g)]\frac{\partial }{\partial \sum }\left(\mu +\sum^{\text{T}}q(\epsilon )\right). \end{array}$$

Taking the derivatives of the expressions means,

(45) $$\frac{\partial }{\partial \sum }\text{ln}\;p(D|g[\lambda ,\epsilon ])={\left[d-L(g)\right]}^{\text{T}}\left(\frac{\partial L}{\partial g}\right)q(\epsilon ).$$

This expression can be rearranged using the relationship *A*^T^*BC* = *C*^T^*B*^T^*A*, such that,

(46) $$\frac{\partial }{\partial \sum }\text{ln}\;p(D|g[\lambda ,\epsilon ])=q{\left(\epsilon \right)}^{\text{T}}{\left(\frac{\partial L}{\partial g}\right)}^{\text{T}}[d-L(g)].$$

Finally, sampling from the standard normal distribution *ϵ*^*i*^ ~ *q*(*ϵ*), we have

(47) $$\frac{\partial }{\partial \sum }p\left(D|m^{i}\right)={\left(\epsilon^{i}\right)}^{\text{T}}{\left(\frac{\partial L}{\partial m^{i}}\right)}^{\text{T}}\left[d-L\left(m^{i}\right)\right].$$

This gradient provides the update for the standard deviation. Therefore the iterative process to obtain the standard deviation is,

(48) $$\sum =\sum -{\left(\epsilon^{i}\right)}^{\text{T}}{\text{Δ}}_{m}.$$

Because uncertainty is equal to the variance of the distribution, we take the square of the standard deviation image (Σ^2^) once the iterative process has completed.

In the statistical model above, the mean-field approximation is used to simplify the description. However, researchers often have an interest in the covariance of the model parameters. We will propose a model-based approach to this in section 5.3, but it is also possible to specify a full Gaussian for the approximating distribution (equation (37)). This is done by enumerating the off-diagonal elements of the variance matrix (also called the covariance matrix), and then the usual derivation can be followed as above.

### 3.3 The adjoint-state method and stochastic variational inference

The Fréchet derivatives *∂L*/*∂g* in equations (41) and (46) are computationally expensive because a numerical simulation of the wavefield propagation must be run for each model parameter *g* in turn. In practice, FWI uses an adjoint-state method to make the gradient computationally tractable. Now, we will show how the adjoint-state method can be applied to the standard deviation gradient in equation (46).

Starting with the expression for the forward problem in equation (2),

$$\varsigma \left(x_{\text{s}}\right)=H(m)u_{\text{p}}(m)$$

take the derivative of both sides with respect to *m* and rearrange,

(49) $$\frac{\partial u_{\text{p}}{\left(m\right)}^{\text{T}}}{\partial m}=u_{\text{p}}{\left(m\right)}^{\text{T}}\frac{\partial H(m)}{\partial m}{\left[H{\left(m\right)}^{-1}\right]}^{\text{T}}.$$

Note that $\frac{\partial L(m)}{\partial m}=\frac{\partial u_{\text{p}}\left(m,x_{\text{r}}\right)}{\partial m}$ . Then, using the probabilistic notation (*q*(*m*; λ) = *g*[λ, *ϵ*]) and inserting equation (49) into the standard deviation update (equation (46)) yields,

(50) $$\frac{\partial }{\partial \sum }p(D|g[\lambda ,\epsilon ])=q(\epsilon )u_{\text{p}}{\left(g\right)}^{\text{T}}\frac{\partial H(g)}{\partial g}{\left[H{\left(g\right)}^{-1}\right]}^{\text{T}}[d-L(g)].$$

In addition, we can define a new wavefield, called the adjoint wavefield, by rearranging equation (2) such that,

(51) $$u_{\text{adj}}(m)={\left[H{\left(m\right)}^{-1}\right]}^{\text{T}}\varsigma_{\text{adj}}\left(x_{\text{r}}\right).$$

Using the probabilistic notation, defining an adjoint source *d − L*(*g*) = *ς*_adj_(*x*_r_), and applying the stochastic operator *H*(*g*)^−1^ to the source, the adjoint wavefield *u*_adj_(*g*) can defined as,

(52) $$u_{\text{adj}}(g)={\left[H{\left(g\right)}^{-1}\right]}^{\text{T}}[d-L(g)].$$

Finally, using this definition it becomes apparent that the last two terms in equation (50) represent a new wavefield propagating from the receivers to the source,

(53) $$\begin{array}{c} \frac{\partial }{\partial \sum }p(D|g[\lambda ,\epsilon ])=q{\left(\epsilon \right)}^{\text{T}}u_{\text{p}}{\left(g\right)}^{\text{T}}\frac{\partial H(g)}{\partial g}u_{\text{adj}}(g)\\ =q{\left(\epsilon \right)}^{\text{T}}{\text{Δ}}_{m} \end{array}.$$

This is called the adjoint wavefield because it has terminal conditions, which are the difference between the observed and predicted wavefield data at the receivers, and is calculated by backwards time-stepping [23]. Therefore the Fréchet derivatives in equations (41) and (46) can be calculated by calculating the zero-lag correlation between the predicted wavefield and the adjoint wavefield, and it is computationally efficient to do this at each time-step in a backwards time-stepping scheme.

Algorithm 1 **Full-waveform inversion.**

**Input:** *m*, ς, *x*_s_, *x*_r_

**Returns:** *m*

1: **while** *n* ⩽ 76 **do**
2:     *L*(*m*) = **forward**(*m, ς, x*_s_, *x*_r_) (equation (2))
3:     $\text{Γ}=∥d-L(m){∥}_{2}^{2}$
4:     Δ_*m*_ = **adjoint**(*m, ς, x*_s_, *x*_r_, Γ) (equation (54))
5:     *m* ← *m* + Δ_*m*_

Algorithm 2 **Full-waveform inversion using SVI.**

**Input:** *µ*, Σ, *ς, x*_s_, *x*_r_

**Returns:** *µ*, Σ

1: **while** *n* ⩽ 76 **do**
2:     *ϵ*^*i*^ ~ 𝒩 (0, *I*)
3:     *m*^*i*^ = *µ* +∑ · *ϵ*^*i*^
4:     *L*(*m*) = **forward**(*m*^*i*^, *ς, x*_s_, *x*_r_) (equation (2))
5:     $\text{Γ}=∥d-L(m){∥}_{2}^{2}$
6:     Δ_*m*_ = **adjoint**(*m*^*i*^, *ς, x*_s_, *x*_r_, Γ) (equation (54))
7:     *µ* ← *µ* + Δ_*m*_
8:     Σ ← Σ+ *ϵ*^(*i*)^ · Δ_*m*_

A similar analysis finds that the adjoint-state equation for the mean in equation (41) is,

(54) $$\begin{array}{c} \frac{\partial }{\partial \mu }p(D|g[\lambda ,\epsilon ])=u_{\text{p}}{\left(g\right)}^{\text{T}}\frac{\partial H(g)}{\partial g}u_{\text{adj}}(g)|\\ ={\text{Δ}}_{m}. \end{array}$$

This derivation demonstrates that the adjoint-state method calculation, required for the gradient with respect to the mean (equation (54)), can be recycled when calculating the gradient with respect to the standard deviation (equation (53)). This means that the computational cost of the standard deviation gradient is minimal.

The mean-field Gaussian pathwise derivative estimator is also simple to implement. Algorithm 1 outlines the practical aspects required to implement standard FWI and algorithm 2 outlines the practical aspects required to implement SVI–FWI. The major changes between SVI–FWI and standard FWI are highlighted in red. It is apparent that SVI–FWI requires three simple changes to the standard FWI approach. The important changes are in line 3, where a sample from the distribution over the model parameters in introduced, and line 8, where the update for the variance is implemented.

### 3.4 Set-up of experiment and simulation

#### 3.4.1 The in vitro dataset

**Figure 1:**
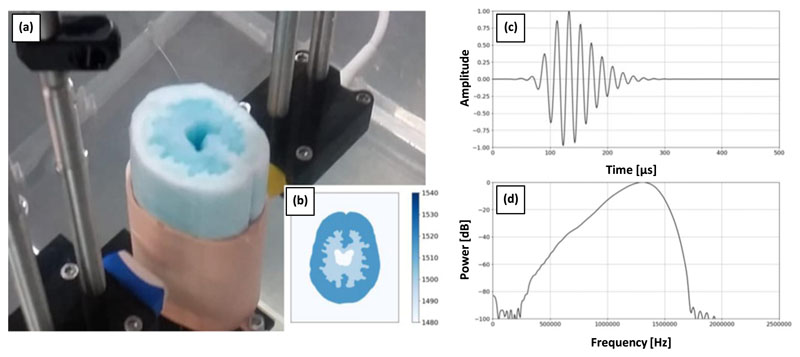
(a) The ultrasound tomography apparatus with the PVA tissue-mimicking phantom (centre) surrounded by two P4-1 transducers which are held mechanically in suspension from two rotary motors. (b) The design-image used to produce the moulds for the phantom. The transmitted source wavelet is shown in the time (b) and frequency domains.

Figure 1 shows the poly-vinyl acetate (PVA) phantom used for the *in vitro* experiment. The phantom was produced using moulds, manufactured from the design-image shown in figure 1(b). The reconstructions were compared to the design-image using the structural similarity (SSIM) metric [29] to assess their accuracy. The phantom has a width 60.5(mm), a length 78.8(mm) and depth 130(mm). The phantom consists of two layers with different sounds speed [30], which do not vary in depth. Thus the sound-speed varies in the imaging plane but is invariant out of plane. This 2.5D layout prevents out-of-plane effects superimposing on the 2D reconstruction plane. The transducers rotate in a circle with diameter 150(mm) centred on the centre of the PVA phantom.

The wavefield data were acquired using two P4-1 transducers (ATL, USA), which have 96 elements and were held mechanically in suspension from two rotary motors. Figure 1(a) shows this set-up, with the two transducers on opposite sides of the PVA phantom. Each transducer can be rotated to one of 16 possible positions around the phantom and, during a single acquisition, one of the transducers act as a source while both transducers act as a receiver. The source has a centre frequency of 1.4(MHz), its excitation signal is shown in figure 1(c) and its frequency content is shown in figure 1(d).

A common cause of reconstruction failure in FWI is cycle skipping. This occurs when the observed and predicted wavefield data arrive more than 90° out-of-phase and causes the reconstruction to fail. Multi-scale methods are a popular way to prevent cycle-skipping [31], and work by low-pass filtering the observed and predicted wavefield data until the pass-band produces filtered data that are in-phase. Therefore, *in vitro* data are low pass filtered at 700(kHz) to prevent cycle-skipping and 1(MHz) to induce it.

In total there are 384 wavefield data acquisition sequences because 24 sources are used for each of the 16 source positions. A source is a single element on the source transducer. There are 16 possible transducer positions which are located between 0° and 337.5° in 22.5° steps. For a single acquisition sequence, the source transducer is moved to one of the 16 positions and fixed in place. The receiver transducer is then moved to one of 11 positions. For example, if the source position is at 0°, the 11 receive positions are between 45° and 315° (in 22.5° steps). The four remaining positions (two either side of the source transducer) are left unoccupied to prevent the transducers colliding. The source fires the excitation signal and the wavefield data are collected at both the source and receive transducer. Then, the receive transducer is moved to another position and the source is fired again (with data collected at both transducers again). Once the receiver has moved to all 11 positions, the acquisition sequence for that source is complete. Now the source changes, either another of the 24 elements at the current position, or one of the 24 elements at a new position is used. Wavefield data are acquired for each of the 384 sources, at which point the full acquisition process is complete.

This constitutes the data acquisition process for the *in vitro* data-set. This data-set is used to demonstrate image quality changes due to progressive decoding (i.e. a resolution improvement), reconstruction failure, and a source position artefact. To demonstrate the sensitivity of the estimator to image quality issues caused by discrepancies between the true physics and the physics of the inverse problem, an *in silico* data-set is used.

#### 3.4.2 The *in silico* dataset

This data-set consists of 510 single element receivers and 510 sources. The sources and receivers are arranged around a circle of diameter 50 (mm) and sound-speed 1540 (m s^−1^). The circle is embedded in a background with sound speed 1500 [m s^−1^], which is roughly equivalent to water. The source excitation signal has a centre frequency of 370 (kHz) and the inversion proceeds after the data are low-pass filtered using a pass-band of 700 (kHz). Observed data were simulated for two different cases. First, when the true physics and the inverse physics match, the density of the circle is 1010 (g cm^−3^), which is the same as the background. Second, when the true physics does not match the inverse physics, the observed data is simulated for a circle with a density of 1220 (g cm^−3^).

The inverse problem assumes that density is homogeneous. Therefore, in the correct-physics case, this assumption is met. In the incorrect-physics case, the change in density at the boundary of the circle violates this assumption. The result is increased reflection and reduced transmission compared to the homogeneous case.

#### 3.4.3 The inversion algorithm

The inversion was performed using in-house FWI software written in Python using Pytorch. The software used a finite-difference time domain wave equation solver. For the *in vitro* case, a 890 × 891 grid with a grid-spacing of 0.2 (mm) and a time-step of 0.038 (*µ*s) was used. For the *in silico* case, a 200 × 200 grid with a grid-spacing of 0.5 (mm) and a time-step of 0.06 (*µ*s) was used. The numerical simulations were found to be stable, with limited dispersion, when using a Laplacian approximation which was second-order in time and tenth-order in space. The accuracy of the propagator was assessed by comparing simulated against *in vitro* observations of wavefield data for water, where the speed of sound of water was measured using a time-of-flight measurement. For the *in vitro* data, the algorithm was considered to have converged after 76 iterations.

## 4 Results: uncertainty as a measure of image quality

To demonstrate our claim of no-cost uncertainty estimation, we measured the computation time of the mean estimator (equation (54)) and the standard deviation estimator (equation (53)) during the *in vitro* reconstruction. Table 1 shows the time required to execute the mean and standard deviation update on a CPU (Ryzen Threadripper 2950X 16 core, Advanced Mirco Devices, CA) and a GPU (Quadro RTX 8000, Nvidia, CA). Compute time is an average of ten iterations.

In the case of the GPU, the standard deviation update takes approximately *one nine thousandth* of the computation time of the mean update. This difference is more pronounced in the case of the CPU because the compute time for the mean update is longer, but the standard deviation update is shorter. We believe this is because of the time taken for transfer data between the CPU and GPU, which negates the parallel computing power of the GPU. It is clear that the uncertainty estimator has effectively no computational cost.

As previously introduced (in section 3), the mean-field Gaussian uncertainty estimator is not a true representation of the pixel-wise variation of the mean. However, this estimator does show useful behaviours. Specifically, we demonstrate with the following results that the estimator can be used for progressive decoding, to detect reconstruction failures, artefacts, and to identify physical modelling discrepancies.

### 4.1 Progressive decoding

**Figure 2:**
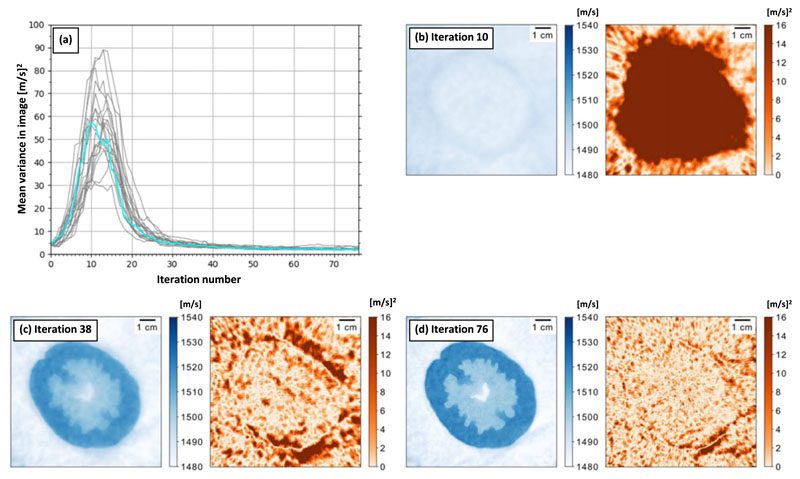
Reconstructions using SVI–FWI and the *in vitro* dataset, low pass filtered at 700 (kHz). (a) The average variance per iteration for 20 different reconstruction processes. (b)–(d) Examples of the mean (left) and variance (right) reconstructions corresponding to the process indicated by the cyan line in (a).

Figure 2 shows results for an inversion of the *in vitro* data-set following low-pass filtering at 700 (kHz). Figure 2(d) shows the reconstruction after 76 iterations. The SSIM was found to be 0.83, indicating a high degree of similarity between the design-image and the final reconstruction. Figure 2(a) demonstrates the stability of the uncertainty estimator by repeating the reconstruction process 20 times and plotting the average of the variance per reconstruction per iteration for the 76 iterations. This shows that for 18 runs, the variance consistently converges to a variance of approximately 2 (m s^−1^). Note the two runs with the highest mean variance (approximately 3 (m s^−1^)) contain artefacts which are discussed in section 4.3.

Descriptively, figure 2(a) shows that the average variance undergoes a rapid increase, followed by a similar decline, and then a consistent improvement until convergence after 76 iterations. The average variance for the reconstruction process presented in figures 2(b)–(d) is indicated in cyan. Figure 2(b) shows that by iteration ten the mean has not reconstructed to a plausible sound speed. This corresponds to high variance of 63.34 (m s^−1^)^2^ on average, which is localised where the mean is incorrect. Figure 2(c) shows that by iteration 38 the mean reconstruction is approximately correct but blurred. The uncertainty estimate is significantly reduced to 3.74 (m s^−1^)^2^ on average. Figure 2(d) shows the reconstruction by iteration 76, the mean is much sharper and the average variance has again reduced to 2.07 (m s^−1^)^2^.

Interestingly, the variance reconstruction in figure 2(c) contains low-frequency correlated noise and the variance in figure 2(d) contains high frequency correlated noise. This is related to the covariance between the pixels. Covariance is an extension of the concept of variance; instead of measuring the variation of a single pixel around its mean, covariance measures the common variation between two pixels. The mean-field estimator assumes that the pixels are uncorrelated and therefore have no covariance. The presence of correlated noise indicates a failing in this assumption, and the length-scale of the correlation could indicate the length-scale of the missing correlations.

### 4.2 Detecting reconstruction failure

**Figure 3:**
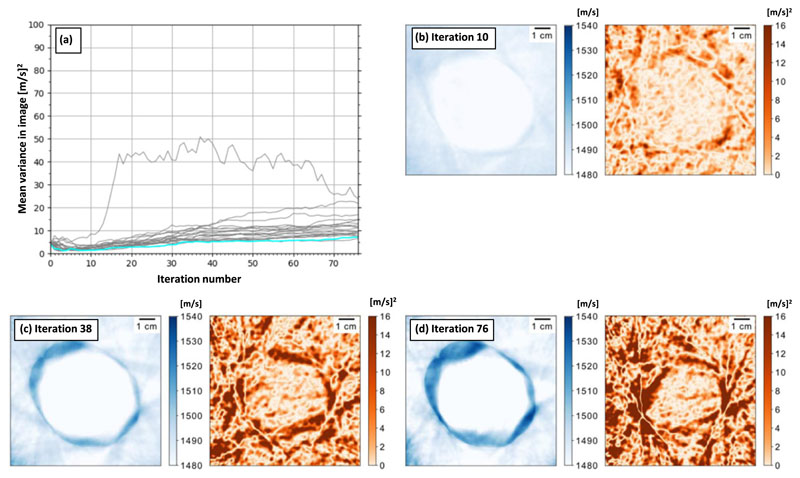
Reconstructions using SVI–FWI and the *in vitro* dataset, low pass filtered at 1 (MHz). (a) The average variance per iteration for 20 different reconstruction processes. (b)–(d) Examples of the mean (left) and variance (right) reconstructions corresponding to the process indicated by the cyan line in (a).

Figure 3 shows results for the inversion of the *in vitro* data-set following low-pass filtering at 1(MHz). Figure 3(d) shows the reconstruction has failed to reconstruct the expected sound-speed image. The SSIM was found to be 0.41, indicating a low degree of similarity between the design-image and the final reconstruction. In figure 3(a) the variance starts low, but increases consistently as the reconstruction process progresses. This is significantly different to the 700 (kHz) case. The average variance for the reconstruction process presented in figures 3(b)–(d) is indicated in cyan. Figure 3(b) shows that by iteration 10, the mean estimate of the sound speed has reduced. Figure 3(c) shows that by iteration 38, the mean estimate remains poor and the variance estimate has increased significantly. Figure 3(d) shows that the variance continues to increase as the number of iterations increases to 76. Note that figure 3 shows reconstructions which were selected from the trend-line with the lowest average variance, the final variance is higher for the other 19 reconstruction processes.

After iteration 76, the variance of the 20 repeats at 1 (MHz) are consistently higher than the equivalent 700 (kHz) examples. In addition, the mean reconstruction in figure 3(d) is indicative of cycle-skipping, which is a known cause of reconstruction failure in FWI [20]. Finally, comparing figure 2(a) with figure 3(a) shows that the average of the variance is more unstable in the 1 (MHz) case. The average variance in these figures is also trending in different directions; in the 1 (MHz) case the variance increases regularly, while it decreases in the 700 (kHz) case. Therefore, these plots show that uncertainty is consistently higher and increasing when the *in vitro* data is cycle-skipped.

### 4.3 Artefact detection

**Figure 4:**
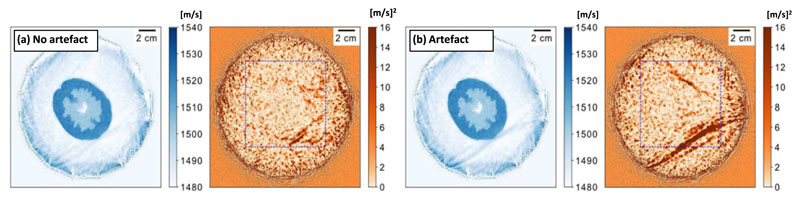
(a) An uncropped replica of the result in figure 2(d), where the crop-box is indicated by the blue dotted square. (b) Another example reconstruction produced using the 700 (kHZ) filtered data, but in this case the posterior reconstruction contains an artefact due to source positioning error.

In figure 2(a) two of the reconstruction processes produce a higher average variance than the others. Figure 4(b) shows one of these two runs. Figure 4(a) is a repeat of figure 2(d), but now the reconstruction has not been cropped. The blue boxes in figure 4 indicate the crop used in figures 2 and 3.

It is clear from figure 4(b) that the high average variance is caused by a single region. When we compare the mean reconstruction of figure 4(b) to the mean reconstruction in figure 4(a), it is clear that the region with high variance corresponds to an artefact. This artefact is caused by an intermittent source–receiver positioning error (see section 5.1.3 for details).

### 4.4 Wave equation physics

**Figure 5:**
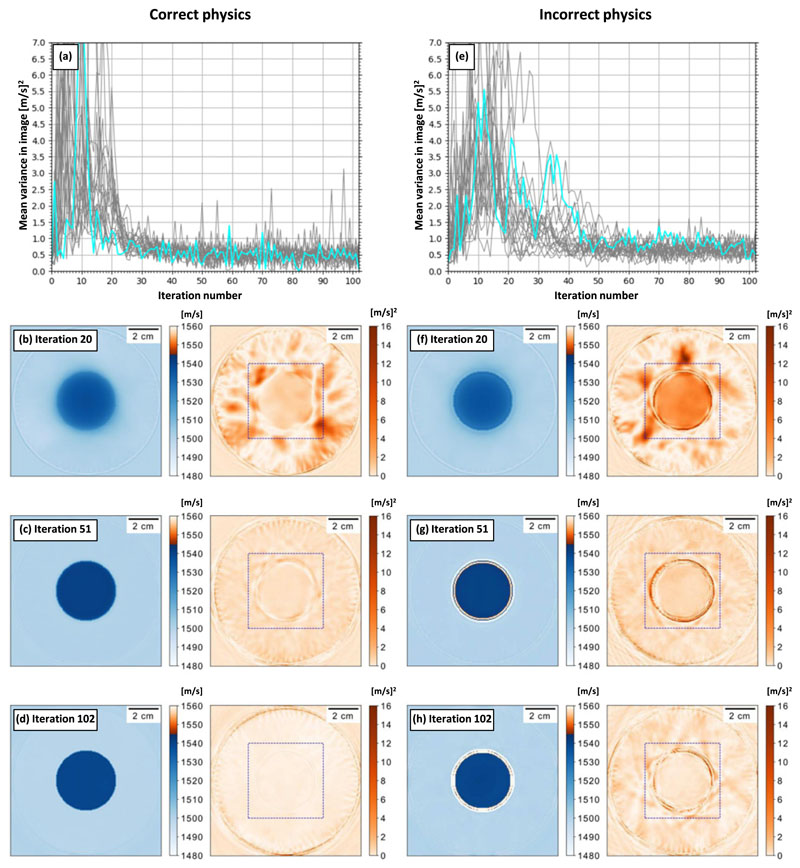
An *in silico* demonstration of the effect of physics on the reconstruction process. Reconstructions (blue) of a circle are created assuming density is homogeneous. In (a)–(d) this is correct, and the observed data are created assuming a constant density of 1010 (g cm^−3^). In (e)–(h) this is incorrect because the circle has density 1220 (g cm^−3^), but surrounding water has density 1010 (g cm^−3^). When the homogeneous assumption is inaccurate, the variance (orange) is higher.

Our FWI adjoint method (i.e. the reconstruction process) assumes homogeneous density. However, figure 5 shows two cases. First, when the physics of the FWI adjoint method is correct; in other words, the reconstruction truly has homogeneous density and therefore the circle has the same density as the surrounding water. Second, when the physics of the FWI adjoint method is incorrect and, therefore, the density of the reconstruction region is inhomogeneous. In the homogeneous case, the circle has a density of 1220 (g cm^−3^) but the surrounding water has a density of 1010 (g cm^−3^).

Figures 5(a) and (e) show behaviour characteristic of an accurate reconstruction process; the variance rises, falls, and settles to a low value once all the data from all the sources have been used. However, comparing figures 5(a) and (e) shows that the mean variance of the reconstructions is higher and more unstable when there is a mismatch between the physics of the FWI adjoint method and that of the observed data. By iteration 102 the mean variance is roughly the same in both cases. The relative increase in the variance in figure 5(e) is because the change in density increases the reflections and reduces the transmissions from the circle compared to the homogeneous case assumed for FWI.

After the first 51 iterations, each source has been used once. This corresponds to the reconstructions in figures 5(c) and (g). Comparing these figures shows that the adjoint method explains the increased reflection and reduced transmission due to the inhomogeneous density by increasing the sound speed at the boundary of the circle (e.g. see figure 5(g)). This also creates a ring of high variance in the boundary region. When the adjoint method is true to the physics of the observed data (in figure 5(c)), it is apparent that the sound speed artefact and high variance are no longer present.

Figure 5(h) shows that the variance artefact decreases without completely disappearing. This happens because the high sound speed at the boundary explains the mismatch between the physics of the inversion and data. Figure 5(d) has the lowest average variance and the sound speeds in the mean reconstruction correspond to the true values.

## 5 Discussion: context, utility, and extensions

SVI was developed to stabilise back-propagation through variational auto-encoder architectures which are based on artificial neural networks [25–27]. SVI has since been applied to BNN’s [28], where each node in a layer is parameterised by a Gaussian distribution, and Gaussian processes [32], which assume a Gaussian distribution of functions. Similarly, this paper describes a Gaussian process which uses the wave-equation as the link function between the sound speed (i.e. the parameter space) and the wavefields (i.e. the function space). There are also connections between our approach and BNN’s, because neural networks and numerical differential equation solvers are connected [33, 34].

The ability to recycle the adjoint method in equations (53) and (54) proves that the meanfield Gaussian variance estimator is computationally inexpensive. This is because the additional vector multiplication is computationally efficient. Measurement of the computation time for the mean and standard deviation gradients support this finding and show that the estimator has a negligible cost compared to solving the FWI adjoint method. Indeed, this shows that the mean-field Gaussian estimator is substantially less computationally expensive than the Hessian calculation or the ensembling technique. It also has a smaller memory footprint than BNNs. Finally, our results demonstrate that the parameters of an optimisation process using the pathwise derivative can be tailored such that the inversion process is consistently stable using *in silico* and *in vitro* observed data. This is an advantage over the stochastic gradient Langevin dynamics method, which requires a step-size schedule that is difficult to tune [19].

### 5.1 Comments on the results

#### 5.1.1 Progressive decoding

The results in figure 2 demonstrate that the mean-field variance estimate is a useful metric for measuring the resolution of the reconstructed image during an iterative reconstruction process. It is significant that this result is achieved using an experimental *in vitro* data-set because it demonstrates the fundamental strength of our approach, namely, SVI is immediately applicable to tomographic reconstruction (and adjoint methods) without requiring additional effort to work with real data. It is also unclear whether similar robustness will be achieved by the alternative methods discussed previously, many of which have only been demonstrated on synthetic data.

#### 5.1.2 Detecting reconstruction failure

The mean-field Gaussian estimator is a clear indicator of reconstruction failure. When a mean reconstruction is produced from cycle-skipped data, the variance reconstruction is a significantly higher than the case where the mean reconstruction converges correctly.

The results suggest that while average variance is useful, the local variance is less reliable for detecting cycle-skipping. This effect is a result of the mechanics of cycle-skipping. The cycle-skipping appears to cause the reconstruction to diverge, meaning that the mean update (equation (54)) consistently reduces the value of the sound speed in the centre of the reconstruction. When the multiplication operation is applied to obtain the variance update (equation (53)), approximately half the samples have their direction reversed because sampling the standard normal distribution produces roughly equal quantities of positive and negative samples (the mean of the standard normal distribution is 0). Because the mean updates are consistently negative in the centre of the reconstruction, then roughly half the variance updates are positive and, therefore, the variance update is small. A lack of covariance information might also be hindering the quality of the estimate in this region. Figure 3(b) shows that in the early iterations the sound speed of the entire reconstruction reduces simultaneously, which means that the covariance between pixels would be high. When the direction of the mean update at the edge of the phantom becomes positive, the magnitude of the variance update also increases locally. If the covariance between the outer and inner parts of the phantom were initially high, this could cause the variance in the central region to increase.

This line of reasoning also leads to an interesting interpretation of figure 2. Initially the mean update is far from the ground truth, which causes some of the samples from the model distribution to be cycle-skipped while others are not. In turn, this causes the variance to increase rapidly in the region where cycle-skipping occurs, this is observed in figure 2(b). The variance will reduce as the mean reconstruction converges, because now most of the samples from the model distribution occur in the locally-convex part of the objective function, this is observed in figure 2(c). The general trend shown in figure 2(a) also follows this pattern.

#### 5.1.3 Artefact detection

Our results indicate that locally high variance estimates can indicate artefacts. We expect the detection of artefacts to be consistent regardless of their presentation. In addition to the detection of the source position artefact *in vitro*, figure 5 shows that a non-obvious artefact due to an error in the reconstruction physics can also be found. We believe that the robustness of the detection is due to artefacts being driven by epistemic errors between the gradients produced from different source–receiver pairs. Above, we explain that high variance is caused by epistemic errors in the data (e.g. cycle-skipping) so, where these gradients conflict, high variance is inevitable.

#### 5.1.4 Wave equation physics

The variance estimator is sensitive to perturbations in the density which are not explained by the FWI modelling physics. The average variance is consistently higher when the simulated data contains the effects of variable density and the regions of high variance are localised to the regions where the sound speed is erroneous (i.e. the high sound speed at the boundary). In addition, we expect that the failure of reconstruction physics approximations will drive high variance *in vivo*. For example, the magnitude of the density perturbation is reasonable for human tissue; it is greater than the variation between different brain tissues, but smaller than the difference between cortical bone and cerebrospinal fluid [35].

### 5.2 Usefulness

In many applications, MCMC is considered to be the most effective method to estimate uncertainty. However, in high dimensional problems, MCMC algorithms are often numerically intractable. We have developed an uncertainty estimator which assumes a mean-field Gaussian distribution over the model parameters. This approach is simple, very computationally efficient, and will accessible in many problems where uncertainty is not usually estimated.

It is appropriate to note that the mean-field Gaussian approximation of the posterior relies on a simplistic approximation, but this approximation means that the posterior can be calculated quickly and efficiently. Accurate-but-intractable estimators such as MCMC are also flawed, but in a different way. Furthermore, sections 2.1 and 3 show that the approximations associated with the mean-field Gaussian estimator are consistent with the approximations used by many gradient-based solvers. Therefore, the mean-field Gaussian uncertainty estimates may be accurate for a variety of different problems. Thus, we propose that uncertainty estimates should be generated where possible and evaluated based on the information that they can provide to a particular problem.

To illustrate the potential of our estimator, we demonstrate some potential applications to image quality assessment in FWI tomography. Comparisons with other methods are avoided because different approximations are appropriate for different problems and their effects are difficult to anticipate. In future, a comparative study will be performed because is be important to quantify the accuracy of the estimator for specific applications. However, findings of such a study will not hold generally, meaning an application specific quantitative study might be misleading.

#### 5.2.1 Progressive decoding

A measurement of the resolution (or sharpness) of the reconstruction has a number of important applications. For example, FWI is a computationally expensive reconstruction algorithm which is slow to converge but, with the variance estimator, it is possible to adapt the stopping point after achieving sufficiently high reconstructed resolution or, equivalently, sufficiently low average variance. This is the simplest way to implement a dynamic reconstruction algorithm. Another application is to provide preliminary reconstructions alongside an estimate of the resolution of the current image. This is useful because a clinician can be presented with an intermediate result which has a low resolution and they can identify larger features in the image. Then, the final result can be presented when available.

During their treatment, a patient may be monitored using a variety of different imaging systems that achieve different image qualities. Variance estimates can simplify comparisons between reconstructions by quantifying resolution and identifying artefacts. For example, an objective measure of resolution makes it possible to make volume measurements with specific error bounds and the pixel-wise estimate means variation in resolution across the image can be taken into account.

#### 5.2.2 Detecting reconstruction failure

The capability to automatically identify reconstruction failure enables several applications. First, many imaging systems have settings which need to be optimised in order to improve the system performance. The pass-band of the low-pass filter for multiscale FWI is one such example. With the variance estimate, it is possible for a non-expert to set the pass-band frequency without relying on subjective judgement. Second, our method also enables error detection for each reconstruction. Unexpected issues, such as the presence of metallic implants, might cause cycle-skipping or obstruction in part of the reconstruction. With our method, the pass-band can be tailored until the reconstruction performs correctly, or the reconstruction can be conclusively identified as having failed. We expect the detection of cycle-skipping to be particularly useful in geophysics.

#### 5.2.3 Artefact detection

Artefacts are an important problem in medical imaging. In tomography, classic examples of artefacts are metallic streaking artefacts in x-ray computed tomography, and movement artefacts in magnetic resonance imaging. Figure 4 illustrates an intermittent source position artefact in USCT and figure 5 shows a density induced artefact. Artefacts obstruct parts of the image, meaning that they reduce diagnostic capability by reducing the visibility of important features. Artefacts can be subtle, meaning that they can mislead clinicians and lead to false positives or measurement errors (i.e. they can distort an image by changing the magnitude, shape, or size of the feature of interest). Worse, automatic diagnosis by artificial intelligence methods can be severely misled by artefacts, and artefacts are challenging to detect by pure feature detection because they have a wide variation in their presentation. The robust detection of artefacts will become increasingly important as automatic diagnosis becomes more common in medical imaging.

#### 5.2.4 Wave equation physics

All tomographic reconstruction algorithms rely on approximations to the imaging physics. Section 4.4 shows that uncertainty can be used to monitor regions of the reconstruction where these approximations are failing. This is important as the failure of these reconstructions is a cause of artefacts and it is useful to be able to identify these. It is also possible that reconstruction algorithms using different physics approximations could be compared using the variance estimator, meaning that the effect of the different approximations can be identified objectively.

#### 5.3 Future work

In addition to the comparative study already discussed, this paper has a number of important extensions. Most prominent, is to investigate the use of prior information in the reconstruction problem. Interestingly, this compliments another topic, which is to incorporate covariance information into the reconstruction. One approach to estimate correlation it to assume a parametric relationship between the pixels of the reconstructed image (e.g. assuming a full Gaussian distribution). However, this approach is likely to reduce the quality of the variance estimate by enforcing nonphysical correlations between pixels. For example, a fully-specified Gaussian tends to prevent sharp changes in contrast such as the change between the skull and the brain. An alternative approach is to use a non-parametric method and learn the correlation function during the reconstruction process. The archetypal non-parametric method is a convolutional artificial neural network, suggesting that combining our pathwise derivative approach with the DIP could be interesting. Prior information is naturally incorporates into a model-based framework because the correlations must be learnt from a database of example reconstructions *a priori*. Thus we will study the data-based priors to correctly solve Bayes’ equation and to regularise ultrasound tomography of the brain.

For FWI of brain tissues, another important direction is to evaluate the ability of the estimator to monitor the effect of elastic wave propagation. This is a promising area of further study because elastic wave-propagation is present in bone tissues. We expect that our uncertainty estimator will be more sensitive to the discrepancies of this type because elastic effects modulate the phase of the wavefield data, FWI is more sensitive to phase changes, and, unlike density, elastic effects cannot be consistently explained by adjusting the sound speed. It will also be important to assess whether the estimator is equally effective for 3D imaging because most modern reconstruction algorithms produce 3D posteriors.

Finally, the variance reconstruction is available per-iteration, meaning that the estimates could be used to produce adaptive or dynamic reconstruction algorithms, where the optimal choice of data is identified for the next gradient calculation. In FWI, this would consist of choosing the best source–receiver pairs in order to adjust the model parameters where the variance is highest. In MRI, the very low cost of the variance estimates means on-line reconstruction might be possible. Hence, the variance could be used to guide the data acquisition process. A particularly useful implementation could be to combine these estimators with reinforcement learning in order to achieve these aims.

## 6 Conclusion

Most uncertainty estimators are too numerically expensive to solve for high-dimensional problems. In this paper, an estimator has been developed which will be numerically tractable in almost all inverse problems. The estimator is developed using SVI, which solves Bayes’ equation using a gradient-based method. If mean-field Gaussian approximations to the posterior and likelihood are used, a *MAP* estimator can be obtained which is comparable to the gradient-based optimisation of an data-fit (or objective) function formulated as an L2-norm. In addition, we have shown that solving the data-fit function with respect to the variance of the posterior is essentially free. The mean-field Gaussian approximation to the posterior is a strong assumption but these assumptions are not more aggressive than standard gradient-based optimisation which chooses the L2-norm. In addition, we have illustrated the usefulness of this estimator for image quality assessment in FWI tomography. FWI is a non-linear problem, so we expect that other non-linear problems may also benefit from the use of this estimator. Because of its low computational cost and demonstrated similarity to traditional gradient-based optimisation, we expect that our variance estimator will have similar utility in other fields which also rely on adjoint methods.

## Figures and Tables

**Table 1:** The time taken to compute the mean and standard deviation update on different devices.

Hardware	Mean update (s)	Standard deviation update (s)
CPU	1200 ± 90	0.003 56 ± 0.000 04
GPU	122 ± 1	0.014 ± 0.004

## Data Availability

The data that support the findings of this study are available upon reasonable request from the authors.
